# Psychological Status, Compliance, Serum Brain-Derived Neurotrophic Factor, and Nerve Growth Factor Levels of Patients with Depression after Augmented Mindfulness-Based Cognitive Therapy

**DOI:** 10.1155/2022/1097982

**Published:** 2022-01-04

**Authors:** Huirong Guo, Yuming Ren, Bailing Huang, Junru Wang, Xuhuang Yang, Yali Wang

**Affiliations:** ^1^Department of Psychiatry, The First Affiliated Hospital of Zhengzhou University, Zhengzhou, Henan Province 450052, China; ^2^Office of Academic Studies, Xinxiang Medical University, Xinxiang, Henan Province 453003, China; ^3^Department of Psychiatry, People's Hospital of Gongyi City, Gongyi, Henan Province 451299, China

## Abstract

**Objective:**

Mindfulness-based cognitive therapy (MBCT) is a cost-effective psychosocial program that prevents relapse/recurrence in major depression. The present study aimed to analyze the effects of augmented MBCT along with standard treatment dominated by pharmacotherapy on psychological state, compliance, brain-derived neurotrophic factor (BDNF), and nerve growth factor (NGF) expression levels in patients with depression.

**Methods:**

A total of 160 eligible patients with depression in The First Affiliated Hospital of Zhengzhou University were included in this study. The study randomly assigned the patients to the experimental group (*n* = 80) and control group (*n* = 80). All participants were assessed with the questionnaires including the 17-item Hamilton Depression Rating Scale (HAMD-17), Rosenberg Self-esteem Scale (RSES), Self-Acceptance Questionnaire (SAQ), and Stigma Scale (Scale of Stigma in People with Mental Illness, SSPM). The serum levels of BDNF and NGF were detected by enzyme-linked immunosorbent assay (ELISA).

**Results:**

After 8 weeks of treatment, the experimental group showed significant lower HAMD-17 score, higher RSES, and SAQ score, as well as lower SSPM score compared with the control group (*P* < 0.01). Furthermore, ELISA revealed that the serum levels of BDNF and NGF remarkably increased in the experimental group after treatment (*P* < 0.001).

**Conclusions:**

Our data showed that augmented MBCT combined with pharmacotherapy contributed to improvement on patients' psychological state, compliance, and disease recurrence.

## 1. Introduction

Depression, also known as depressive disorder, is the most common psychiatric symptom with high prevalence. Its consequences were well established without appropriate treatment [1]. The patients in serious condition are confronted with a higher risk of recurrence, leading to life quality and social function impairment [2]. Depression not only severely affects patient's own life and work but also harms the physical and mental health of patients' families [3] and causes great pressure on the society [4]. There has been more than 300 million people experiencing depression worldwide, and the socioeconomic burden of this debilitating disorder is anticipated to increase remarkably over the coming decades against a background of increasing global turmoil [5]. Adult patients with major depression were mainly given acute treatment with a standard antidepressant pharmacotherapy, psychotherapy, or their combination [6]. The effectiveness of pharmacotherapy for depression has been confirmed, while the remission rate ranges from 25% to 35%. Therefore, it is essential to start new treatment to enhance response rate and reduce recurrence rate [7].

Mindfulness-Based Cognitive Therapy (MBCT) is group-based psychological intervention that mainly uses mindfulness training and cognitive behavioral techniques to eliminate depression symptoms in clinical and nonclinical populations. It has been confirmed as an effective way to prevent relapse in major depressive disorder [8]. In the late 1970s, MBCT was developed by Kabat-Zinn at the University of Massachusetts Medical Center. MBCT cultivates nonjudgmental awareness of the present moment, allowing people to be aware the bodily sensations, feelings, and thoughts, which was founded by Kabat-Jinn at the University of Massachusetts Medical School in the late 1970s [9]. It is a method of using Buddhist meditation to help patients deal with stress, pain, and illness. Mental training has become the most widely used decompression training therapy in the American medical system, and it is also extensively applied to medical treatment in other Western countries such as Canada and United Kingdom. In addition to its effectiveness in preventing depression recurrence, MBCT has been found to reduce fear of recurrence and anxiety in women after diagnosis and treatment of ovarian cancer [10]. Brain-derived neurotrophic factor (BDNF) is a neurotrophin that plays an important role in neuron survival, growth, and maintenance in key brain circuits referring to emotional and cognitive function [11]. Accumulating evidence shows that neuroplastic mechanisms dependent of BDNF are deleteriously changed in major depression and animal models of depressive disorder [12]. Nerve Growth Factor (NGF) has been discovered as a signaling molecule that is vital to survival, protection, and differentiation of sympathetic and peripheral sensory neurons [13]. Declined NGF serum levels can be served as a possible biomarker of major depression disorder [14]. The present study aimed to analyze the effects of augmented MBCT combined with standard treatment (mainly pharmacotherapy) on depression patients about their psychological state, compliance, BDNF, and NGF expression levels.

## 2. Methods

### 2.1. Participants

From October 2016 to October 2019, 160 patients with depression in The First Affiliated Hospital of Zhengzhou University were included in the study. The study randomly assigned the patients to the experimental group (*n* = 80) and control group (*n* = 80). All the patients were known to have depression from the diagnostic result of the International Classification of Diseases, 10th Revision (ICD-10) codes for depression [15]. These patients aged from 18 to 60 years, were educated above junior high school, got the 17-item Hamilton Depression Scale (HAMD-17) score above 17, and had no history of administration of psychotropic drugs within 2 weeks. Those patients must be excluded if they met any of following criteria: (a) presence of organic brain diseases; (b) severe physical diseases; (c) drug allergy; (d) suicide attempt; (e) mental retardation; (f) excessive drinking; and (g) women during pregnancy or lactation. The patients in the control group were given oral administration of escitalopram, and those in the experimental group received MBCT in addition to oral administration of escitalopram. Written informed consent was obtained from each participant or their guardians. The study protocol was approved by the Ethics Committee of The First Affiliated Hospital of Zhengzhou University.

### 2.2. Treatment Protocols

Two groups of patients were initially treated with escitalopram (10 mg/tablet, 5 mg/d), and the dose was adjusted to 10 to 20 mg/d within 2 weeks for 8 weeks. None of the patients used other antidepressants or mood stabilizers or antipsychotic drugs during treatment. Those with sleep disorders can appropriately take nonbenzodiazepines zolpidem or zopiclone. The patients in the experimental group received MBCT (once a week, 120 min/time, 8 times in total) during escitalopram treatment. MBCT was performed and modified as previously described by Segal et al. [16]. Briefly, the experimental group was divided into 5 subgroups, with 12 persons per group. After receiving the exactly same MBCT each time, each subgroup was required to do additional homework of meditation practices. The well-experienced psychotherapists were specifically trained and involved in the treatment to ensure intervention process consistency of each subgroup. The protocols of MBCT were in accordance with the work of Ma and Teasdale [17]. The concrete scheme was as follows: at the first phase (1-2 times), the psychotherapists introduced the neurophysiological mechanisms of depression and emphasize the importance of continuous treatment. Each subgroup practiced mindfulness through eating raisins and guided body scans; the second phase (3-4 times) included basic practices including mindfulness walking, 3-minute breathing spaces and focused awareness on daily activities, and expanding practices to mental activities consisting of thoughts and emotions; and the third phase (5–8 times) is all about objective perception of correlation among events, thoughts and feelings, and acceptance of any thoughts and emotions.

### 2.3. Outcome Measures

Before treatment and after 8 weeks of treatment, the relevant questionnaires were performed to evaluate patients' condition. The HAMD-17 is a multiple-choice questionnaire with 17 items for evaluating symptoms of major depression such as depressed mood, guilt, suicide agitation, and loss of weight [18]. The Rosenberg Self-esteem Scale (RSES) [19], Self-Acceptance Questionnaire (SAQ) [20], and Stigma Scale (Scale of Stigma in People with Mental Illness, SSPM) were used to assess patients' mental state and compliance. A well-trained nurse who did not participate in the study conducted the survey and guided the patients to fill the questionnaires with unified guidelines. After clear explanation, the patient shall fill in the form in sequence and submitted the questionnaire immediately. . The internal consistency coefficient of the scale evaluation is more than 0.85. Fasting venous blood (5 ml) were obtained from each subject before and after treatment, and the blood sample was centrifuged at the speed of 2000 r/min for 10 min at 4°C. The serum levels of BDNF and NGF were determined by enzyme-linked immunosorbent assay (ELISA) methods using commercially available kits (R&D system, USA).

### 2.4. Statistical Analysis

All data were input into the database through Epidata3.1 software. SPSS18 statistical software was used to perform data analysis. Statistical methods included statistical description, an independent-sample *t* test, a paired-sample *t* test, and a chi-square test. The difference was statistically significant with *P* < 0.05.

## 3. Results

### 3.1. Demographic Characteristics of Included Patients with Depression

The experimental group consisted of 27 males and 33 males, with an average age of (37.48 ± 11.89) years, an average education of (14.02 ± 6.11) years, and an average disease duration of (2.59 ± 2.03) years. The experimental group consisted of 29 males and 31 males, with an average age of (37.03 ± 11.74) years, an average education of (13.88 ± 6.75) years, and an average disease duration of (2.47 ± 1.98) years. There were no statistically significant differences in gender distribution, age, years of education, and disease duration between the experimental group and control group (*P* > 0.05).

### 3.2. Augmented MBCT following Pharmacotherapy Attenuated Depressive-Like Symptoms

There was no significant difference in HAMD-17 scores between the two groups before treatment (*P* > 0.05). The HAMD-17 after treatment in the experimental group and the control group was significantly different compared with that before treatment (*P* < 0.01). After 8 weeks of treatment, the HAMD-17 score of the experimental group was lower than that of the control group, and the difference was statistically significant (*P* < 0.01). The data are detailed in Table 1.

### 3.3. Augmented MBCT following Pharmacotherapy Improved the Self-Esteem Level of Depression Patients

There was no significant difference in SES score between the two groups of patients before treatment (*P* > 0.05). After 8 weeks of treatment, the results of the SES in the experimental group were higher than those before treatment, and the difference was statistically significant (*P* < 0.01). The SES score of patients in the control group after 8 weeks of treatment was higher than that before treatment, and the difference was statistically significant (*P* > 0.05). After 8 weeks of treatment, the SES score of the experimental group was higher than that of the control group, and the difference was statistically significant (*P* < 0.01). The data are detailed in Table 2.

### 3.4. Augmented MBCT following Pharmacotherapy Improved the Self-Acceptance of Depression Patients

There was no statistically significant difference in the SAQ score between the two groups before treatment (*P* > 0.05). The SAQ score of patients in the experimental group after 8 weeks of treatment was higher than that before treatment, and the difference was statistically significant (*P* < 0.01). The SAQ score of patients in the control group after 8 weeks of treatment was higher than that before treatment, and the difference was not statistically significant (*P* > 0.05). After 8 weeks of treatment, the SAQ score of the experimental group was higher than that of the control group, and the difference was statistically significant (*P* < 0.01). The data are detailed in Table 3.

### 3.5. Augmented MBCT following Pharmacotherapy Reduced the Stigma Level of Depression Patients

Before treatment, there was no statistically significant difference in the SSPM scores between the two groups of patients (*P* > 0.05). After 8 weeks of treatment, the results of the SSPM in the experimental group were lower than those before treatment, and the difference was statistically significant (*P* < 0.01). The SSPM scores of the patients in the control group after 8 weeks of treatment were lower than those before treatment, and the difference was statistically significant (*P* < 0.01). After 8 weeks of treatment, the SSPM score of the experimental group was lower than that of the control group, and the difference was statistically significant (*P* < 0.01). The data are detailed in Table 4.

### 3.6. Augmented MBCT following Pharmacotherapy Elevated the Serum Levels of BDNF and NGF in Depression Patients

**Figure 1:**
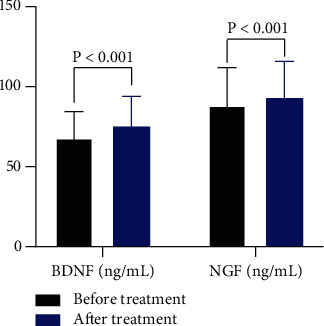
The serum levels of BDNF and NGF in patients with depression before and after MBCT.

Before treatment, the serum levels of BDNF and NGF in patients with depression were 68.18 ± 16.37 (ng/mL) and 75.44 ± 18.59 (ng/mL), respectively. After MBCT, the serum levels of BDNF and NGF in patients with depression were 88.47 ± 23.65 (ng/mL) and 93.29 ± 22.62 (ng/mL), respectively. Results of ELISA revealed that the serum levels of BDNF and NGF were remarkably increased in patients with depression after MBCT (*P* < 0.001, Figure 1).

## 4. Discussion

Patients with major depression had negative emotions such as sadness, irritability, reduced self-esteem, and even suicidal tendency. MBCT based on cognitive therapy is developed through mindfulness meditation intervention. It has been widely used in current psychological counseling practice. It is known that mindfulness refers to the patient's awareness state and characteristics of the present experience, and mindfulness training refers to the process of paying attention to open and free consciousness, including the formation of awareness and thinking and the purpose of implementation. The main purpose is to guide the patient to perceive and accept the current experience in a nonjudgmental attitude so that the patient can form an experience of emotion, thinking, and feeling. The relief of clinical symptoms is the primary goal of antidepressant treatment, but the elimination of symptoms does not mean the end of treatment for depression. Patients with depression still have obvious psychological and social function damage, but these problems have not received enough attention for a long time. Psychosocial function mainly refers to the ability of individuals to perform their roles in the social environment. Patients with depression will show a series of psychosocial function impairments (such as work, life, and interpersonal communication), accompanied by a significant decline in the quality of life [21]. Antidepressant treatment failed to effectively improve psychosocial impairment [22]. This means the psychosocial function of patients with depression is more complicated than the clinical symptoms. Combined psychotherapy has a significant effect on solving the abovementioned problems [23]. Therefore, the combination of traditional medication intervention and appropriate psychological treatment contributed to improve patients' clinical condition and psychosocial function. Different attitude can alleviate or increase the stress caused by life events. Depression patients tend to use negative attitude to deal with life events, which leads to an adverse impact on the prognosis of the disease [24]. After 8 weeks of treatment with drugs and MBCT, the SAQ score of the experimental group was significantly higher than that of the control group. MBCT emphasizes the observation of self-experience, emotion, or behavior with an open and tolerant attitude, so as to promote patients to form a more comprehensive cognition and evaluate themselves more positively [25]. The results of this study showed that, after 8 weeks of treatment, the HAMD-17 scores of the experimental group and the control group were significantly different than those before treatment. Furthermore, the HAMD-17 score of the experimental group was significantly lower than that of the control group. The results of the SES in the experimental group were remarkably higher than those before treatment The SES score of patients in the control group after 8 weeks of treatment was statistically higher than that before treatment, and the experimental group showed much higher SES score than the control group The SAQ score of patients in the experimental group after 8 weeks of treatment was higher than that before treatment, and the difference was statistically significant. The SAQ score of patients in the control group after 8 weeks of treatment was obviously higher than that before treatment, and the SAQ score in the experimental group was significantly higher than that of the control group After 8 weeks of treatment, the results of the SSPM in the experimental group and the control group were remarkably lower than those before treatment, and the experimental group indicated significantly lower score of SSPM than the control group. Additionally, the study suggested that augmented MBCT following pharmacotherapy elevated the serum levels of BDNF and NGF in depression patients. BDNF and NGF levels are usually used to reflect the effectiveness of antidepressant treatment or psychotherapy [26].

The current study provides several clinical and research implications, indicating that MBCT might have a beneficial effect on other important factors except depressive symptoms in the patients with depression. MBCT combined with pharmacotherapy contributes to improve patients' psychological state and compliance and increases the serum levels of BDNF and NGF preventing the recurrence of depression.

## Figures and Tables

**Table 1:** Comparison of the scores of the HAMD-17 scale between the experimental group and control group before and 8 weeks after treatment.

Group	Before treatment	After treatment	*t* value	*P* value
Experimental group	25.76 ± 4.59	9.25 ± 3.82	17.58	<0.001^*∗*^
Control group	26.23 ± 5.12	10.98 ± 4.21	13.25	0.001^*∗*^
*t* value	−0.35	−2.54		
*P* value	0.59	0.008^*∗*^		

^*∗*^, *P* < 0.01.

**Table 2:** Comparison of the scores of the RSES scale between the experimental group and control group before and 8 weeks after treatment.

Group	Before treatment	After treatment	*t* value	*P* value
Experimental group	19.82 ± 4.24	28.69 ± 5.56	−8.01	<0.001^*∗*^
Control group	20.13 ± 4.65	22.86 ± 5.19	−2.61	0.020^#^
*t* value	0.32	5.69		
*P* value	0.87	<0.001^*∗*^		

^*∗*^, *P* < 0.01 and ^#^*P* < 0.05.

**Table 3:** Comparison of the scores of the SAQ scale between the experimental group and control group before and 8 weeks after treatment.

Index	Before treatment		After treatment		Comparison between groups after treatment	
	Experimental group	Control group	Experimental group	Control group	*t* value	*P* value
Self-acceptance	19.86 ± 2.56	19.55 ± 2.65	21.46 ± 2.58	19.59 ± 2.51	2.78	0.006^*∗*^
Self-evaluation	18.45 ± 1.22	18.65 ± 1.58	18.59 ± 1.98	18.91 ± 2.19	2.04	0.045^#^
SAQ score	38.56 ± 5.23	37.88 ± 4.16	41.36 ± 4.01	38.49 ± 4.24	2.58	0.005^*∗*^

^*∗*^, *P* < 0.01 and ^#^*P* < 0.05.

**Table 4:** Comparison of the scores of the SSPM scale between the experimental group and control group before and 8 weeks after treatment.

Group	Before treatment	After treatment	*t* value	*P* value
Experimental group	41.36 ± 15.68	21.89 ± 5.26	8.25	<0.001^*∗*^
Control group	40.28 ± 15.31	32.56 ± 12.59	2.58	0.005^*∗*^
*t* value	0.18	−5.26		
*P* value	0.89	<0.001^*∗*^		

^*∗*^, *P* < 0.01.

## Data Availability

The data used to support the findings of this study are included within the article.
